# Mr. Dawes, on the Œconomy of the Liver

**Published:** 1807-10

**Authors:** John Dawes

**Affiliations:** Member of the Royal College of Surgeons in London. Birmingham


### Medical and Phyfical Journal.

VOL. XVIII.] OCTOBER, 1807. [NO. 104.

*Printed for R. PHILLIPS, by W. Thorne, Red Lion Court, Fleet Street, London*

### *To the Editors of the Medical and Phyfical Journal.*

GENTLEMEN,

THE variety of hypotheses which have been formed, to account for the important function of the Liver; and the manner in which the blood is distributed to this organ being not analogous to that in any other organ in the human body, render this subject one of the most curious in physiological inquiry.

Secretions in general are formed from arterial blood; and most frequently the artery which supplies the secreting viscus with blood for its nourishment, supplies also an extra quantity for its secreting function. This, however, is not the order of circulation through the liver. The hepatic artery is supposed to supply it with nourishment, and the vena portarum with blood, from which it secretes bile. This is probably true. But the properties ascribed to this venous blood, and the various arguments made use of to demonstrate those properties, are somewhat vague. It cannot easily be admitted, that after it has given to other viscera its best properties, the blood should then be fit for a secretion of as much importance as any other in the animal economy. That such blood does pass through the vena portarum is admitted: and that bile is secreted from blood which passes through the vena portarum is also admitted; therefore the conclusion at present is, that "venous blood is more favourable to this secretion than arterial;" and, "that whatever changes are induced on the blood in passing from the arterial to the venous condition, those changes furnish the principles which adapt the blood more completely to this purpose." This is a conclusion if not upon wrong, at least upon doubtful premises; and the error seems to be supported by our ignorance of the real use of the spleen. The human mind hesitates sitates upon admitting a principle so contrary to the general law of Nature, therefore, to support the favourite hypothesis, it calls in the assistance of the spleen to furnish the peritoneal venous blood with a "putrescent and consequently diluting liquor," to make a mass, said to be the most proper for the secretion of bile.

These arguments are certainly not demonstrative, nor are they supported by a single fact. Let us, therefore, change the position, and instead of venous putrescent blood, allow the liver, blood for secretion as pure as any other secreting viscus, and endeavour to demonstrate the fact upon those premises. Let us suppose that *the liver secretes bile from arterial blood.*

That bile *may* be secreted from arterial blood, few will deny, that have read Mr. Abernethy's case in the Philosophical Transactions. In this case, the vena portarum did *not* enter the liver; and it is not very improbable that Nature may supply the liver with arterial blood for secretion, through means that have hitherto eluded observation. The spleen, perhaps, is the viscus through whose mediation the liver may be so supplied: its anatomy, in my opinion, favours this conclusion; it is spongy and elastic, and therefore well calculated to act as a reservoir of arterial blood, for the use of the liver: it has no excretory duct, therefore probably it secretes nothing *from* the blood. This opinion is further confirmed by an experiment of Dr. Saunders,* which was successfully made for another purpose. But it is an experiment well calculated to

---

* Treatise on the Liver, Chap. iii. § 10. EXPERIMENT.

"The *abdomen* of a living dog being opened, and the spleen with its vessels being drawn gently out, blood was taken both from the artery and vein, and received into cups of similar shape and equal size. On weighing such, there was found to be 420 grains of arterial, and 468 of venous blood. Both coagulated in less than two minutes, and in about the usual time they separated into *serum* and *crassamentum*. In 24 hours, the *serum* of both was accurately weighed: the 420 grains of blood from the splenic artery separated 191 grains of serum; the 468 grains from the vein separated 213 grains.

§ 11 "But our conceptions of this matter will be much assisted by instituting a comparison with one common standard, still preserving the *ratio*.

"Therefore we say, 1000 parts of blood from the splenic artery separated 454, while the same quantity from the vein yielded 455: a difference so inconsiderable as this surely can never be laid hold of as a proof that the spleen is subservient to the liver, on the principle of a diluting organ.

to prove that the blood, passing from the spleen by the splenic vein, is *arterial* blood.

Things equal to one and the same thing, are equal to one another; the blood from the splenic vein is as much arterial as that from the artery, excepting the $\frac{1}{455}$ part, which might arise from the demand of the spleen itself for its own nourishment.

A question here naturally presents itself. If the spleen supply the liver with arterial blood for the secretion of bile, what use can be assigned for the venous blood, which passes through the vena portarum from the chylopoietic viscera? an use, apparently, of no small importance.— The texture of the liver being composed almost wholly of blood vessels, and very inelastic, seems to require a *constant* stream of blood to support its *bulk* and *figure*, and thereby prevent the danger of its rupture; the venous blood of the chylopoietic viscera seems well adapted for this mechanical purpose.

Again, how is the circulation in the fœtal liver to be accounted for, the principal part of which is *arterial* blood from the umbilical vein? The answer to this question is founded upon a fact, that secreting glands act in proportion to the demand of the constitution for the secreted liquor: the salivary glands, for instance, pour out an immense quantity of saliva during the stimulus of mastication, but very little at other times, when not excited to action. The liver of the fœtus secretes but little bile, perhaps no more than the gall-bladder will contain for the digestion of the first food; the secreting function of the liver, therefore, seems to be suspended in this state of animal existence; and whether it be *venous* or *arterial* blood that circulates, is in this case of no moment; but as soon as the child is born, or rather as soon as food is taken, the secreting function of the liver is excited to form bile for its digestion; this gland must then, that is, at birth, be perfect in its structure. The increased size of the liver of an infant, in proportion to some other glands, indicates also its early importance. The principal use of the blood of this vein passing through the liver, appears to me to act partly mechanically, in keeping the vessels of the liver distended during their formation, that is, by its pressure against their sides, contributing thus to their formation of a due size or capacity. The arterial quality may also assist the hepatic artery in the nourishment of so large a viscus, and in that way contribute also to its structure. It is also necessarily, for the most part arterial, be-

 cause cause little other can pass through the liver either from the mesenteric or emulgent veins, for no food is taken, and little or no urine is secreted before birth. The ramifications of this vein do not become obliterated after birth, when the vein itself does, but continue to grow, and actually become branches of the vena portarum.

In the adult of the human species, where food is taken after certain intervals, (during health) it is probable that less bile is secreted, in such interval, than when chyme is passing the pylorus.

The distribution of the arteries of the abdominal viscera demands particular attention, and when duly considered, will perhaps be found to favour this hypothesis. The cæliac, the superior and inferior mesenteric arteries, are three azygic branches, principally destined for the abdominal viscera. The first, or cæliac, almost immediately divides itself into three trunks or branches; the first of which is distributed to the stomach, called the coronary artery; the second, or splenica, to the spleen; and the third, or hepatica, to the liver. Either of these three branches being excited to increased action must affect the other two, in consequence of being connected by the cæliac, which is equal in capacity to the sum of the three said branches; had each branch been sent off immediately from the aorta, without the intervention of the cæliac, the excitement of one would not have affected the other.

I shall conclude this Inquiry with what appears to me to be the economy of Nature in the process of digestion. As soon as food, mixed with saliva, is received into the stomach, after abstinence, the coronary artery becomes stimulated to supply that viscus with blood, for the secretion of the gastric liquor; the cæliac at the same time partakes of the stimulus, and sends an increased quantity through the splenica to the spleen; and through the hepatica to the liver. This extra quantity sent to the liver is not supposed to be destined for the secretion of bile, but for the purpose of occasionally invigorating the secreting organic structure of the liver: this action, at the same time, elicits the *arterial* blood from the spleen (previously sent by the splenic artery) for the secretion of bile. At this time the first digested portion of food is about to pass into the duodenum, which is another source of stimulus for the liver to exercise its secreting function, the first portion being probably supplied by the gall bladder.

I am, &c.

JOHN DAWES,

Member of the Royal College of Surgeons in London.

*Birmingham, Sept.* 14, 1807.

